# Antimicrobial Drug Interactions: Systematic Evaluation of Protein and Nucleic Acid Synthesis Inhibitors

**DOI:** 10.3390/antibiotics8030114

**Published:** 2019-08-09

**Authors:** Kaan Yilancioglu

**Affiliations:** Department of Chemistry and Biology Engineering, Faculty of Engineering and Natural Sciences, Uskudar University, Uskudar, Istanbul 34662, Turkey; kaan.yilancioglu@uskudar.edu.tr; Tel.: +90-532-652-97-20

**Keywords:** antagonism, antibiotic interactions, synergy

## Abstract

Antimicrobial multidrug resistance and its transmission among strains are serious problems. Success rate is decreased and treatment options are narrowed due to increasing bacterial multidrug resistance. On the other hand, the need for long-term efforts to discover new antibiotics and difficulties finding new treatment protocols make this problem more complex. Combination therapy, especially with synergistic use of antimicrobials is a rational treatment option with huge benefits. Thus, screening antibiotic interactions is crucial for finding better treatment options. Clinicians currently use combinatorial antibiotic treatment as an effective treatment option. However, antibiotics can show synergistic or antagonistic interactions when used together. In our study, we aimed to investigate interactions of antibiotics with different mechanisms of action. Antibiotics, which act as protein synthesis inhibitors (P) and nucleic acid synthesis inhibitors (N) were used in our study. We tested 66 (PN), 15 (NN), and 55 (PP) drug pairs on the *Escherichia coli* strain. The Loewe additivity model was used and alpha scores were calculated for analysis of interactions of drug combinations. Drug interactions were categorized as synergistic or antagonistic. Accordingly, pairwise combinations of protein synthesis inhibitors (PP) showed stronger synergistic interactions than those of nucleic acid synthesis inhibitors (NN) and nucleic acid synthesis–protein synthesis inhibitors (PN). As a result, the importance of mechanisms of action of drugs is emphasized in the selection of synergistic drug combinations.

## 1. Introduction

Antimicrobial resistance and transmission of drug resistance complicate management of infections. Thus, hospitalization and mortality rates are increasing. Moreover, antimicrobial therapy becomes even more complicated due to limited treatment options [1].

In order to prevent and overcome emerging drug resistance, antimicrobial combination therapy is a rational approach. Synergistic interactions among drugs provide a huge benefit for treatment. However, some risks should be considered, since some of the antibiotics might have antagonistic interactions. Therefore, drug interactions should be carefully evaluated.

Bacterial infections are estimated at over 250 million infections per year and they are causing around $1.6 billion in economic losses each year. Emerging antimicrobial resistance and transmission of resistance among the strains complicates the management of the infection and it increases the rate of hospitalization and mortality. In addition, among patients with underlying secondary diseases antimicrobial drug therapy is even more complicated [2].

Combination therapies based on antibiotic interactions may be used to slow down resistance evolution and increase the potency of drugs. Two or more drugs can interact to result in a different efficacy compared to the independent use of a single drug. If the interaction results in a decreased efficacy, the drugs show antagonism. An increase in efficacy as a result of drug interaction is called synergy. Antagonistic drug pairs gained relevance in the field of antibiotic resistance, as resistant colonies were shown to be selected against when treated with hyper-antagonistic antibiotic combinations [3] and it has been suggested that the risk of evolution of resistance to both drugs may be decreased in antagonistic pairs [4]. Synergistic drug pairs can enhance therapeutic value and overcome toxicity by allowing lower doses [5]. Despite giving resistant cells a selective advantage, synergy may also help prevent the evolution of drug resistance by minimizing the clearance rate of infection, when competition for resources is weak [6]. Another important prevention method is avoiding transmission of resistant pathogenic bacteria. Thus, hand washing is the simplest, cheapest, and the most powerful method. Disinfection of tools and environment is another powerful prevention method against transmission of pathogenic bacteria [7,8,9].

In this study, we aimed to evaluate interactions of the combinations of protein and nucleic acid synthesis inhibitors which are shown in Table 1 with mechanisms of action. Understanding the importance of mechanisms of action of drugs in antibiotic interactions is crucial for better selection of drug combinations. Studies of screening interactions of pairwise combinations of nucleic acid synthesis and protein synthesis inhibitors will shed light on finding more effective drug pairs in therapy.

## 2. Materials and Methods

Bacterial Strain and Culture: *E. coli* is one of the most abundant bacteria in human flora and the potential of its infection risk is high. Thus, model bacteria ATCC 10798 (Lambda+), *E. coli*, were used in this study. Glycerol stocks were prepared and stored at −20 °C. Bacteria were plated on LB (Luria–Bertani) agar for colony formation prior to preparation of the starter cultures. Colonies were picked and grown in LB overnight for MIC (Minimum Inhibitory Concentration) and interaction experiments.

Study Design: Drug interaction experiments were done by using a 4 × 4 checkerboard assay [10]. Accordingly, the concentration of each drug used in the assay increased gradually in each axis with a starting drug concentration of zero (no drug). Protein and nucleic acid synthesis inhibitor drugs are the main classes for treatment of pathogenic bacteria and it is important to find potent synergistic pairs between these two main antibiotic classes in order to have better treatment options. Therefore, these two main classes were selected for the study. Bacteria were grown in LB media and treated with the combinations of eleven protein synthesis and six nucleic acid synthesis inhibitor antibiotics shown in Table 1. End-point optical density (OD, 600 nm) measurements using a 96-well microplate reader were done at the end of overnight incubation.

In drug interaction experiments, each individual drug was used at the concentration that was >50% inhibition at the highest dose and <50% inhibition at the lowest dose, as detailed in a previous study [10,11]. All drugs were purchased from Sigma. Simple two-fold dilution assay was used for MICs for each drug shown in Table 1.

Statistics: Interactions were quantified based on the isophenotypic growth contour method described in Cokol et al. [2] based on the Loewe additivity model [12]. The growth contour was linear for drugs that were non-interacting. Depending on the concavity or convexity of the growth contour, interactions were classified as synergistic or antagonistic. The Null hypothesis according to Loewe’s interaction model was that a drug was non-interacting with itself. Deviations from this null model meant either synergy or antagonism.

Statistical analysis was performed with SPSS version 20.0 for Windows. Comparisons of alpha scores of drug combinations were performed with the Mann—Whitney U test between groups. A value of *p* < 0.05 was considered statistically significant.

## 3. Results

Pairwise combinations of the eleven protein and six nucleic acid synthesis inhibitor antibiotics, with MIC values shown in Table 1, were used in in order to evaluate drug interaction patterns. Combinations of nucleic acid synthesis inhibitor antibiotics (NN) namely, Ciprofloxacin (CIP, 0.05 µg/mL MIC), Levofloxacin (LEV, 0.01 µg/mL MIC), Nalidixic acid (NAL, 8 µg/mL MIC), Trimethoprim (TRI, 1.5 µg/mL MIC), Rifampicin (RIF, 0.5 µg/mL MIC), and 5-Fluorouracil (5-FU, 2 µg/mL MIC), combinations of protein synthesis inhibitor antibiotics (PP) namely, Amikacin (AMK, 13 µg/mL MIC), Gentamicin (GEN, 7 µg/mL MIC), Tobramycin (TOB, 0.7 µg/mL MIC), Tetracycline (TET, 5 µg/mL MIC), Chloramphenicol (CHL, 3.5 µg/mL MIC), Clarithromycin (CLA, 9 µg/mL MIC), Erythromycin (ERY, 15 µg/mL MIC), Fusidic acid (FUS, 80 µg/mL MIC), Spectinomycin (SPE, 2 µg/mL MIC), Roxithromycin (ROX, 0.3 µg/mL MIC), and Mupirocin (MUP, 0.4 µg/mL MIC), and pairwise combinations of both protein synthesis and nucleic acid synthesis inhibitor antibiotics were evaluated. In total, 136 pairwise drug combinations were used which consisted of the 55 PP, 15 NN, and 66 PN antimicrobial combinations shown in Table 2. Statistical analysis demonstrated in Table 2 showed that combinations of protein synthesis inhibitors (PP) were more prone to showing synergistic interactions than those of nucleic acid synthesis inhibitors (NN) and the combination of nucleic acid synthesis–protein synthesis inhibitors (PN). In Table 2, PP–NN, PN–NN, and PN–PP pairwise drug combinations are statistically compared according to their alpha scores in order to demonstrate which class of inhibitors showed a more synergistic or antagonistic manner. According to *p* values, PP combinations were more prone to demonstrate synergistic interactions, as partially shown in previous studies [10].

**Figure 1:**
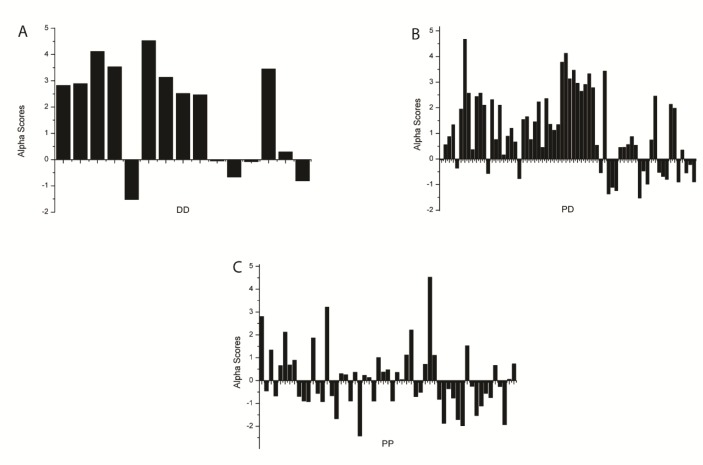
Distributions of alpha values of pairwise drug combinations, which represent synergistic or antagonistic interactions. Negative values show synergistic interactions whereas positive values show antagonistic interactions of combinations of (**A**) nucleic acid synthesis inhibitors (NN), (**B**) combinations of both nucleic acid synthesis and protein synthesis inhibitors (PN), and (**C**) protein synthesis inhibitors (PP).

Figure 1 shows distributions of alpha values of pairwise drug combinations, which represent synergistic or antagonistic interactions. Negative values show synergistic interactions whereas positive values represent antagonistic interactions of combinations of protein synthesis inhibitors (PP), nucleic acid synthesis inhibitors (NN), and combinations of both nucleic acid synthesis and protein synthesis inhibitors (PN). Positive alpha scores or antagonistic interactions are more likely to occur between PN and NN combinations compared to PP drug combinations.

According to statistical analysis demonstrated in Table 2, combinations of protein synthesis inhibitors (PP) showed stronger synergistic interactions than those of nucleic acid synthesis inhibitors (NN) and the combination of nucleic acid synthesis–protein synthesis inhibitors (PN).

## 4. Discussion

Multidrug resistance of bacteria against antimicrobials is a dangerous problem. Combination therapy is a commonly used treatment option especially for serious infections to provide synergistic effects, combat antibiotic resistance, reduce treatment time, and widen the therapeutic spectrum [13].

Drug interactions have become of remarkable scientific interest. Different classes of antibiotics were evaluated in previous studies conducted on different species such as *Klebsiella* sp. [14,15,16]. In this study, 136 pairwise drug combinations, which consisted of 55 PP, 15 NN, and 66 PN antimicrobial combinations were evaluated shown in Table 3. Several studies of synergistic and antagonistic drug interactions of antibiotic combinations in pathogenic species have been published. In comparison with this study, previous results from other studies have shown similar drug interactions. Our results are supported by previous findings. [16,17]. However, there are still a small number of studies in the literature that show categorical and systematic evaluation of antibiotic molecules with different modes of action. There were some studies that showed some of the drug interactions between protein synthesis inhibitors [14,17] in wild-type laboratory strains. Accordingly, our results are in parallel with previous findings of those studies. In our study, combinations of protein synthesis inhibitors tended to be more likely to show synergistic interaction patterns than PN and NN pairwise drug combinations. Chandrasekaran et al. evaluated pairwise drug combinations between 15 different drugs that belong to different antibiotic classes, including cell wall inhibitors. Out of 105 combinations only 14 demonstrated synergistic interactions. In our study synergistic combinations were also found in limited numbers. According to Chandrasekaran et al., out of the 14 synergistic drug interactions found, nearly half were between protein synthesis inhibitors. These findings support this present study. As shown in Table 2, PP drug combinations were more likely to show synergistic drug interactions than NN and PN drug combinations, especially, among protein synthesis inhibitors namely Tetracycline, Chloramphenicol, Erythromycin, and Clarithromycin, which were demonstrated in a similar manner by Chandrasekaran et al.

There are theoretical explanations for drug interaction models. One explanation is that drugs may show synergy if a drug’s action helps a second drug’s availability in the cell. This is called the bioavailability model. According to the bioavailability model, two compounds might form a more potent compound and this would affect different target sites, or a compound might facilitate another compound’s transportation through the cellular membrane [18]. Another explanation is the physical interaction model that two drugs physically interact to make a more potent compound. Another is the same target model that two drugs may be synergistic if they target different sites on the same protein or a target. This suggests that protein synthesis inhibitors, especially those that have the same targets (30S or 50S subunits, etc.) might be more likely to show synergistic interactions. In our study, PP combinations expectedly showed more synergistic interactions, unlike NN combinations. Even if NN combinations had drug pairs with same targets, they did tend to show more additive or antagonistic interactions compared to PP and PN combinations according to statistical analysis between pairwise combinations of different antibiotic classes. This suggests that combinations might have drug pairs with the same targets, but still tend to show antagonistic interactions. According to the drug antagonism model, antagonism occurs when a drug’s action impedes the metabolism of the cell so that the second drug cannot achieve its optimal effect. Some of the antagonistic interactions in our study might be explained by using this model especially for PP and NN combinations.

A systematic exploration of drug interactions was achieved in the context of mechanisms of action of drug molecules. In a previous study, a systematic exploration of antifungal drugs was done. According to the results, antagonistic drug interactions were more likely, compared to synergistic drug interactions [2]. Although, synergistic interactions were seen in limited numbers, our results show that PP combinations are a better choice compared to PN and NN combinations in terms of providing synergistic drug pairs. Mechanisms of action of drug molecules seem to be critical for their potency in combinatorial use [4]. In accordance with the study of Yeh et al., our results clearly show drug classes rule the type of pairwise drug interaction model. In this study, there are interesting points that should be further investigated. Out of eighteen synergistic combinations, eight contained 5-fluorouracil in pairwise combinations. In the PP combinations twelve out of a total of twenty-six synergistic combinations contained a macrolide antibiotic. However, some macrolides did not synergize with some compounds which have similar targets. For instance, AMK is synergistic with CLA, but is not synergistic with ROX or ERY.

Another important discussion point is understanding of the synergistic or antagonistic response in various bacterial genetic backgrounds. In this study, we used the ATCC 10798 strain which has the Lambda+ genotype. This strain might have an SOS response which might be induced especially by nucleic acid synthesis inhibitors such as 5-fluorouracil [19,20]. It is known that induction of the SOS response may induce the lytic phage cycle. This could in turn affect the MIC value of single compound therapy and may affect combinatorial drug treatment. In addition, the lytic cycle requires production of new proteins and DNA. Under treatment with a protein synthesis inhibitor, the lytic cycle might be affected in a combinatorial therapy and drug interaction types may differ.

## 5. Conclusions

In this study, combinations of protein and nucleic acid synthesis inhibitors were evaluated in order to understand their systematic drug interaction patterns. Determination of the mechanisms of action of drug molecules within antibiotic combinations is crucial for better selection of synergistic drug pairs. Detailed mechanistic studies should be conducted with a broader sample size in order to better understand drug interaction patterns.

## Figures and Tables

**Table 1:** List of antibiotics used in the study with minimum inhibitory concentration (MIC) values.

Compounds	Abbreviation	Mechanism of Action	MIC/LB (mg/mL)
Amikacin	AMK	Protein synthesis, 30S inhibition	13
Gentamicin	GEN	Protein synthesis, 30S inhibition	7
Tobramycin	TOB	Protein synthesis, 30S inhibition	0.7
Tetracycline	TET	Protein synthesis, 30S inhibition	5
Spectinomycin	SPE	Protein synthesis, 30S inhibition	2
Clarithromycin	CLA	Protein synthesis, 50S inhibition	9
Erythromycin	ERY	Protein synthesis, 50S inhibition	15
Chloramphenicol	CHL	Protein synthesis, 50S inhibition	3.5
Fusidic acid	FUS	Elongation factor, protein synthesis inhibition	80
Mupirocin	MUP	Isoleucyl transfer, RNA (tRNA) synthetase inhibition	0.4
Roxithromycin	ROX	Protein synthesis, 50S inhibition	0.3
Ciprofloxacin	CIP	DNA gyrase inhibition	0.05
Levofloxacin	LEV	DNA gyrase inhibition	0.01
Nalidixic acid	NAL	DNA gyrase inhibition	8
Trimethoprim	TRI	Folic acid biosynthesis inhibition	1.5
Rifampicin	RIF	RNA polymerase inhibition	0.5
5-Fluorouracil	5FU	Inhibition of the formation of thymidylate from uracil	2

**Table 2:** Mann–Whitney U test for evaluation of alpha scores between different antibiotic groups. NN represents the pairwise combinations of nucleic acid synthesis mechanism-related antibiotics, PP represents the pairwise combinations of protein synthesis mechanism-related antibiotics, and PN represents the pairwise combinations of protein synthesis and nucleic acid synthesis mechanism-related antibiotics. Significance level is <0.05.

Combination Type	N	Min	Q1	Median	Q3	Max
**PP**	55	−2.4	−0.89	−0.25	0.72	4.5
**NN**	15	−1.5	−0.07	2.51	3.45	4.5
	**U**	**Z**	**Prob > |U|**			
	214	−2.8	0.004			
	**N**	**Min**	**Q1**	**Median**	**Q3**	**Max**
**PN**	66	−1.5	−0.25	0.87	2.33	4.67
**NN**	15	−1.5	−0.07	2.51	3.45	4.52
	**U**	**Z**	**Prob > |U|**			
	385	−1.33	0.18			
	**N**	**Min**	**Q1**	**Median**	**Q3**	**Max**
**PN**	66	−1.52	−0.25	0.87	2.33	4.67
**PP**	55	−2.42	−0.89	−0.25	0.72	4.52
	**U**	**Z**	**Prob > |U|**			
	2552	3.83	1.26× 10^−4^			

**Table 3:** Detailed pairwise drug interactions and alpha scores.

DRUG PAIR	ALPHA	1. DRUG	2. DRUG	DRUG PAIR	ALPHA	1. DRUG	2. DRUG
PN	−0.03	AMK	CIP	PP	1.52	AMK	ROX
PN	0.55	AMK	LEV	PP	2.8	AMK	CHL
PN	0.87	AMK	NAL	PP	−0.45	AMK	CLA
PN	1.34	AMK	RIF	PP	1.34	AMK	ERY
PN	−0.35	AMK	TRI	PP	−0.67	AMK	FUS
PN	−0.68	AMK	5FU	PP	0.65	AMK	GEN
PN	1.94	CHL	CIP	PP	2.12	AMK	SPE
PN	4.67	CHL	LEV	PP	0.68	AMK	TET
PN	2.56	CHL	NAL	PP	0.89	AMK	TOB
PN	0.36	CHL	RIF	PP	0.05	AMK	MUP
PN	2.43	CHL	TRI	PP	−0.68	CHL	CLA
PN	−1.52	CHL	5FU	PP	−0.89	CHL	ERY
PN	2.57	CLA	LEV	PP	−0.92	CHL	FUS
PN	2.1	CLA	NAL	PP	1.87	CHL	GEN
PN	−0.56	CLA	RIF	PP	−0.56	CHL	SPE
PN	2.32	CLA	TRI	PP	−0.92	CHL	TET
PN	−0.89	CLA	5FU	PP	3.21	CHL	TOB
PN	2.36	CIP	CLA	PP	−0.26	CHL	ROX
PN	1.35	CIP	ERY	PP	−0.56	CHL	MUP
PN	1.12	CIP	FUS	PP	−0.66	CLA	ERY
PN	1.34	CIP	GEN	PP	−1.67	CLA	FUS
PN	3.78	CIP	SPE	PP	0.30	CLA	GEN
PN	4.12	CIP	TET	PP	0.25	CLA	SPE
PN	2.13	CIP	MUP	PP	−0.89	CLA	TET
PN	3.12	CIP	TOB	PP	0.36	CLA	TOB
PN	3.46	LEV	SPE	PP	−1.11	CLA	MUP
PN	2.95	LEV	TET	PP	−0.25	CLA	ROX
PN	2.64	LEV	TOB	PP	−2.42	ERY	FUS
PN	0.76	LEV	ERY	PP	0.23	ERY	GEN
PN	1.2	LEV	FUS	PP	0.13	ERY	SPE
PN	−0.21	LEV	ROX	PP	−0.90	ERY	TET
PN	0.35	LEV	GEN	PP	1.0	ERY	TOB
PN	2.45	LEV	MUP	PP	−0.81	ERY	MUP
PN	2.1	ERY	NAL	PP	−0.35	ERY	ROX
PN	0.16	ERY	RIF	PP	0.37	FUS	GEN
PN	0.89	ERY	TRI	PP	0.47	FUS	SPE
PN	−0.52	ERY	5FU	PP	−0.89	FUS	TET
PN	0.67	FUS	NAL	PP	0.35	FUS	TOB
PN	−0.76	FUS	RIF	PP	−0.76	FUS	MUP
PN	1.54	FUS	TRI	PP	−1.53	FUS	ROX
PN	0.54	FUS	5FU	PP	2.21	GEN	SPE
PN	1.65	GEN	RIF	PP	−0.70	GEN	TET
PN	0.76	GEN	TRI	PP	−0.51	GEN	TOB
PN	1.98	GEN	5FU	PP	0.04	GEN	ROX
PN	0.45	GEN	NAL	PP	−0.74	GEN	MUP
PN	2.90	NAL	SPE	PP	0.72	SPE	TET
PN	3.32	NAL	TET	PP	4.52	SPE	TOB
PN	2.78	NAL	TOB	PP	−1.87	SPE	ROX
PN	−0.89	NAL	ROX	PP	−1.70	SPE	MUP
PN	0.87	NAL	MUP	PP	1.10	TET	TOB
PN	−1.36	ROX	5FU	PP	−1.98	TET	ROX
PN	−1.10	ROX	RIF	PP	−1.93	TET	MUP
PN	−1.23	ROX	TRI	PP	1.12	ROX	MUP
PN	0.56	ROX	CIP	PP	0.66	ROX	TOB
PN	0.54	RIF	SPE	PP	0.72	TOB	MUP
PN	−0.54	RIF	TET	NN	4.12	5FU	TRI
PN	3.43	RIF	TOB	NN	3.53	5FU	NAL
PN	0.45	RIF	MUP	NN	4.52	5FU	LEV
PN	1.45	TRI	SPE	NN	3.13	5FU	RIF
PN	2.23	TRI	TET	NN	3.45	5FU	CIP
PN	0.45	TRI	TOB	NN	2.46	NAL	CIP
PN	−0.46	TRI	MUP	NN	−0.05	NAL	RIF
PN	−0.98	5FU	SPE	NN	2.82	NAL	TRI
PN	0.74	5FU	MUP	NN	−1.52	NAL	LEV
PN	−0.79	5FU	TOB	NN	−0.07	LEV	CIP
PN	−0.54	5FU	TET	NN	2.89	LEV	RIF
				NN	−0.81	LEV	TRI
				NN	−0.67	TRI	CIP
				NN	0.29	TRI	RIF
				NN	2.51	CIP	RIF
